# TLR2/TLR4-Enhanced TIPE2 Expression Is Involved in Post-Hemorrhagic Shock Mesenteric Lymph-Induced Activation of CD4+T Cells

**DOI:** 10.3389/fimmu.2022.838618

**Published:** 2022-04-29

**Authors:** Hui-Bo Du, Sun-Ban Jiang, Zhen-Ao Zhao, Hong Zhang, Li-Min Zhang, Zhao Wang, Ya-Xiong Guo, Jia-Yi Zhai, Peng Wang, Zi-Gang Zhao, Chun-Yu Niu, Li-Na Jiang

**Affiliations:** ^1^Institute of Microcirculation, Hebei North University, Zhangjiakou, China; ^2^Hebei Key Laboratory of Critical Disease Mechanism and Intervention, Shijiazhuang, China; ^3^Key Laboratory of Microcirculation and Shock in Zhangjiakou City, Zhangjiakou, China; ^4^College of Basic Medicine, Hebei Medical University, Shijiazhuang, China

**Keywords:** hemorrhagic shock, mesenteric lymph, immune dysfunction, CD4+ T lymphocyte, tumor necrosis factor α induced protein 8 like-2

## Abstract

**Purpose:**

Post hemorrhagic shock mesenteric lymph (PHSML) return contributes to CD4^+^ T cell dysfunction, which leads to immune dysfunction and uncontrolled inflammatory response. Tumor necrosis factor α induced protein 8 like-2 (TIPE2) is one of the essential proteins to maintain the immune homeostasis. This study investigated the role of TIPE2 in regulation of CD4^+^ T lymphocyte function in interaction of PHSML and TLR2/TLR4.

**Methods:**

The splenic CD4^+^ T cells were isolated from various mice (WT, TLR2^-/-^, TLR4^-/-^) by immunomagnetic beads, and stimulated with PHSML, normal lymphatic fluid (NML), respectively. Application of TIPE2-carrying interfering fragments of lentivirus were transfected to WT, TLR4^-/-^, and TLR2^-/-^ CD4^+^ T cells, respectively. After interference of TIPE2, they were stimulated with PHSML and NML for the examinations of TIPE2, TLR2, and TLR4 mRNA expressions, proliferation, activation molecules on surface, and cytokine secretion function.

**Results:**

PHSML stimulation significantly upregulated TIPE2, TLR2, and TLR4 mRNA expressions, decreased proliferation, CD25 expression, and IFN-γ secretion, and increased the secretion ability of IL-4 in WT CD4^+^ T cells. TIPE2 silencing enhanced proliferative capacity, upregulated CD25 expression, and increased IFNγ secretion in CD4^+^ T cells. PHSML stimulated TLR2^-/-^CD4^+^ T or TLR4^-/-^CD4^+^ T cells of which TIPE2 were silenced. TLR2 or TLR4 knockout attenuated PHSML-induced CD4^+^ T cells dysfunction; PHSML stimulation of silent TIPE2-expressing TLR2^-/-^CD4^+^ T or TLR4^-/-^CD4^+^ T revealed that the coexistence of low TIPE2 expression with lack of TLR2 or TLR4 eliminated this beneficial effect.

**Conclusion:**

TIPE2 improves the PHSML-mediated CD4^+^T cells dysfunction by regulating TLR2/TLR4 pathway, providing a new intervention target following hemorrhagic shock-induced immune dysfunction.

## Introduction

Death resulted from the loss of blood remains one of the serious threats to humanity worldwide. There are approximately 1.9 million deaths worldwide each year, 1.5 million of which are caused by trauma to the body and consequent bleeding. Hypovolemic shock occurs when severe dehydration or the loss of blood caused by medical malpractice or trauma, which leaves the vascular system with insufficient blood volume and thus severely reduction in perfusion pressure. This hypovolemic shock due to the loss of blood is called hemorrhagic shock (HS) (1). The immune dysfunction caused by HS is an important cause of death in patients. An increasing number of studies have shown that immune dysfunction plays an important role in the development and progression of organ damage and sepsis after shock. The uncontrolled inflammatory response is manifested by systemic inflammation response syndrome (SIRS), compensatory anti-inflammatory response syndrome (CIRS). Immune dysfunction has become an important factor in organ failure after severe shock. Numerous studies have shown that immune dysfunction is associated with organ dysfunction in a variety of diseases and injuries (2–5).

CD4^+^ T lymphocytes are the core cells, which mediate cellular immune responses. TLR on their surface receives exogenous activation signals and is responsible for the secretion of cytokines, which participate in the immune regulatory processes (6–8). T cell-mediated immune regulatory processes are affected by activating signals or inhibitory signals. If the immune regulation is out of control, it will lead to the immune dysfunction, followed by immunosuppression or immune system overactivation (9, 10).

Recent studies have found that the return of the post-hemorrhagic shock mesenteric lymph (PHSML) as toxic product into the circulation is a key mechanism for multi-organ failure and organic immune dysfunction (11–13). Studies have shown that the PHSML causes multiple organ damage by elevating large amounts of cytokines (14). In addition, PHSML has been reported to downregulate the indicators related to the proliferation and activation of CD4^+^ T cells, and induce uncontrollable inflammatory responses, resulting in organismal immune disorders (15). Thus, the studies on PHSML provide a prospect for mitigating or even reversing the harmful effects of HS by regulating inflammation (16).

It is reported that tumor necrosis factor α induced protein 8 like-2 (TIPE2) is essential for the maintenance of immune dynamic homeostasis *in vivo*. It plays an important negative regulatory role in inflammation, as well as the maintenance of immune homeostasis mainly through its effects on T cell receptors (TCR) and Toll like receptor (TLR) signal transduction (17–19). Our previous study found that the intestinal lymphatic pathway plays an important role in the pathogenesis of multi-organ injuries after shock, and that the multi-organ injuries can be attenuated by PHSML draining or reducing its return. Increasing evidences reveal that the downregulation of TIPE2 in CD4^+^ T cells enable the animals resistant to immune disorders by reducing the expression levels of apoptotic genes and immunosuppressive factors (20–22). The TIPE2/TLR4 signaling pathway is also involved in the regulation of macrophage apoptotic process (23), which could be a potential therapeutic target for cellular immune imbalance. In addition, TIPE2-mediated TLR2/TLR4 activation plays an important role in promoting the secretion of inflammatory factors to kill pathogens or reducing the immune response to improve tissue dysfunction (24–26). However, there are no reports on the role of TIPE2 in the activation of CD4^+^ T cells by PHSML. It remains to be further investigated whether silencing TIPE2 could attenuate the immune suppressive effect of PHSML on CD4^+^ T cells and thus the damage of lymphocytes by HS. In this study, we focused on the role of TLR2/TLR4-mediated TIPE2 expression in the activation of CD4^+^ T cells by PHSML.

## Materials and Methods

### Animals

Healthy TLR4^-/-^ and TLR2^-/-^ mice (8-10 weeks) in C57BL/6J background were purchased from Southern Model Biotechnology Co (Shanghai, China). The mice were carefully housed in animal room of Hebei North University, and maintained in temperature (23 ± 2°C), humidity (50 ± 10%) and light (12-h light/dark cycle) controlled animal facilities. The mice had unlimited access to standard mouse chow and clean water. All experimental animals were fasted for 12 hours before surgery and free to water. All experimental animal methods conformed to the animal ethics standards of Hebei North University.

### Reagents

Lentivirus carrying TIPE2-interfering sequences (LV-Tnfaip8I2-RNAi) and negative control virus (CON313) were purchased from Gikai Gene Technology Ltd (Shanghai, China), and CCK8 reagent was purchased from Pulley Gene Technology Ltd (Beijing, China). FITC rat anti-mouse CD4 was purchased from BD Biosciences (USA). APC rat anti-mouse CD3 was purchased from Biolegend (USA). PE rat anti-mouse CD25 was purchased from Biolegend (USA). Immunomagnetic beads for sorting CD4^+^T cells were purchased from Miltenyi (Germany). Anti-TLR2 antibody (209217), Anti-TLR4 antibody (13556) were purchased from Abcam (UK). Anti-TIPE2 antibody (15940-1-AP) was purchased from Proteintech (USA). Mouse anti-β-actin monoclonal antibody (C1313-100) was purchased from Pulley Gene Technology (Beijing, China). Cellular RNA Rapid Extraction Kit, Reverse Transcription Kit, reagents for Real Time Fluorescence Quantitative PCR were purchased from Yishu Biotechnology Co. The primers for TLR2, TLR4 and TIPE2 were designed and synthesized by Biotech Bioengineering (Shanghai, China).

### Hemorrhagic Shock Model

The mice were firstly anaesthetized by inhalation of 3% VOL isoflurane with oxygen flow controlled at 1~1.5 L/min for 1-3 min. Then, the general anesthesia was performed with 1% pentobarbital sodium (50 mg/Kg) injection. The mice were dissected bilaterally in the groin, disinfected with iodine volt and aseptically operated bilaterally in the groin under an operating microscope. The left femoral artery was isolated and cannulated, and 1% heparin sodium solution was injected *via* the femoral artery for systemic anticoagulation. The femoral artery cannula was connected to a PowerLab biosignal acquisition system for real-time monitoring of MAP. And meanwhile, according to the mature method for the preparation of hemorrhagic shock model (27), the right femoral artery was isolated and cannulated, and then a syringe was attached to a microfuge for bloodletting. After the operation was completed and the blood pressure was stabilized, the mice were bled uniformly through the right femoral artery to a MAP of 40 ± 2 mmHg and maintained for 90 min, during which the MAP and status were closely observed. 90 min later, the whole blood bled was equally mixed with Ringer’s fluid for fluid resuscitation in the femoral artery, and the fluid was infused uniformly for 30 min. The Sham group was performed the same operation as the Shock group without bleeding. The Shock group was replicated the hemorrhagic shock model and took the material after 3h. Hemorrhagic shock with PHSML drainage 3 hours was performed in the shock+drainage group.

### Drainage of NML and PHSML

A well-established method in our laboratory was used for drainage and storage of mesenteric lymph. All mice were injected intraperitoneally with a mass fraction of 1% pentobarbital sodium (5 mg/100 g) after general anesthesia for the drainage and collection of NML (normal mesenteric lymph) and PHSML. The cells were removed by cryogenic centrifugation and the supernatant was placed in a -80°C cryogenic refrigerator for use of research.

### Sorting of CD4^+^T Cells

Mice were executed by cervical dislocation. Their spleen tissues were obtained aseptically, then gently ground on a sieve and rinsed continuously with PBS until spleen cell suspension was adequately collected, and single nucleated cells were isolated by lymphocyte isolation solution at a ratio of 2:1. Immunomagnetic bead sorting method (positive sorting) was used to isolate splenic CD4^+^T lymphocytes from various mice (WT, TLR4^-/-^, TLR2^-/-^).

### Lentiviral Transfection of CD4^+^ T Cells

CD4^+^ T cells were isolated from spleen derived from WT, TLR4^-/-^ or TLR2^-/-^ mice. The cells were cultured in RPMI1640 medium containing 10% fetal bovine serum (FBS) (penicillin and streptomycin had been added), and the cell concentration was 5×10^6^ cells/mL. 100 μL of cell suspension was added to 96-well plates precoated with 5 μg/mL rat anti-mouse CD3 and 1 μg/μl rat anti-mouse CD28. WTCD4^+^T, TLR2^-/-^CD4^+^T or TLR4^-/^CD4^+^T cells were transfected with lentivirus carrying TIPE2-specific interfering sequences (shRNA) for 72h. After NML and PHSML stimulation for 24 hours, the cells were collected for subsequent experiments. Lentivirus transfection rate identified by flow cytometry was 82.90% (**Supplementary Figure 1**), which allowed to carry out subsequent experiments.

### CCK8 Assay for Cell Proliferation

CD4^+^ T cells were placed in RPMI1640 medium containing 10% fetal bovine serum, and the cell concentration was adjusted to 2×10^6^/mL. 100 μl of cell suspension per well was added into 96-well plates. After NML and PHSML stimulation for 24 hours, the CCK8 reagent (10 μL) was added to each well. After 3h incubation, the absorbance at 450 nm (OD) was measured by microplate reader.

### Identification and Activity of CD4^+^ T Cell Detected by Flow Cytometry

FITC-conjugated rat anti-mouse CD4 antibody and APC-conjugated rat anti-mouse CD3 antibody added to isolated CD4^+^T cells. Then the purity of the sorted CD4^+^T cells analyzed by flow cytometry was more than 95% (**Supplementary Figure 2**). PE rat anti-mouse CD25 antibody was used to detect the expression of CD25 in CD4^+^T cells of each experimental group by flow cytometry.

### ELISA

The ELISA kit was used to detect the cytokines in the culture supernatant of each experimental group according to the operation instructions, and then the absorbance (OD value) was measured at 450 nm. The contents of IFN-γ, IL-4 and IL-10 in the culture supernatant of CD4^+^ T cells of each experimental group were calculated by the standard curve.

### Quantitative Real-Time PCR (qRT-PCR)

Total RNA was isolated using a rapid extraction kit. And after the purity and concentration were detected to be qualified, DNAse and MIX system were successively added to the quantified RNA in turn, and the reaction was performed at 42°C for 15 min to reversely transcribe RNA into cDNA, which is the reaction template for fluorescence quantitative PCR. The corresponding PCR primers were designed according to the nucleotide sequences of TLR4, TLR2, and TIPE2 to perform fluorescence quantitative assay. The final reaction system consisted of 10uL, with GAPDH as internal reference for template standardization. Quantification of the qRT-PCR results was performed by the 2^−△△CT^ method. The specific primer for amplifying mRNA is listed in **Table 1**.

**Table 1:** Primers for reverse transcription-polymerase chain reaction.

Genes	Primers	Accessionnumber
TIPE2	F:5’-CGATTTCGTCAGAAGCTACG -3’ R:5’-GGGTCAGAGTAGTGATCAAACA-3’	NM_027206
TLR4	F:5’-CCTGTGGACAAGGTCAGCAACTC-3’ R: 5’-CACTCAGACTCGGCACTTAGCAC-3’	NM_021297
TLR2	F: 5’-GACTCTTCACTTAAGCGAGTCT-3’ R: 5’- AACCTGGCCAAGTTAGTATCTC-3’	NM_011905

### Western Blotting

The lysate from CD4^+^ T cells was quantified for protein concentration with the BCA kit. The proteins were separated by 10% SDS-PAGE, and then transferred to PVDF membranes. After blocking with 5% skim milk for 1h, the membrane were incubated with anti-β-actin (1:1000 dilution), anti-TIPE2 (1:1000 dilution), anti-TLR2 (1:1000 dilution) and anti-TLR4 (1:1000 dilution) antibodies overnight. After 3 washes of TBST, the membranes were incubated with secondary antibodies conjugated with horseradish peroxidase (1:8000 dilution). The signal was developed using ECL under ImageQuant LAS 4000 imager. The grayscale values were analyzed with Quantity One software.

### Statistical Analysis

All data were expressed as mean ± standard error (mean ± SE). The experimental data were analyzed by SPSS 23.0 software. Data were compared between multiple groups using one-way ANOVA, and P *<* 0.05 was considered statistically different.

## Results

### Effect of PHSML on the Expression of TIPE2, TLR2 and TLR4 in WT CD4^+^ T Cells

**Figure 1:**
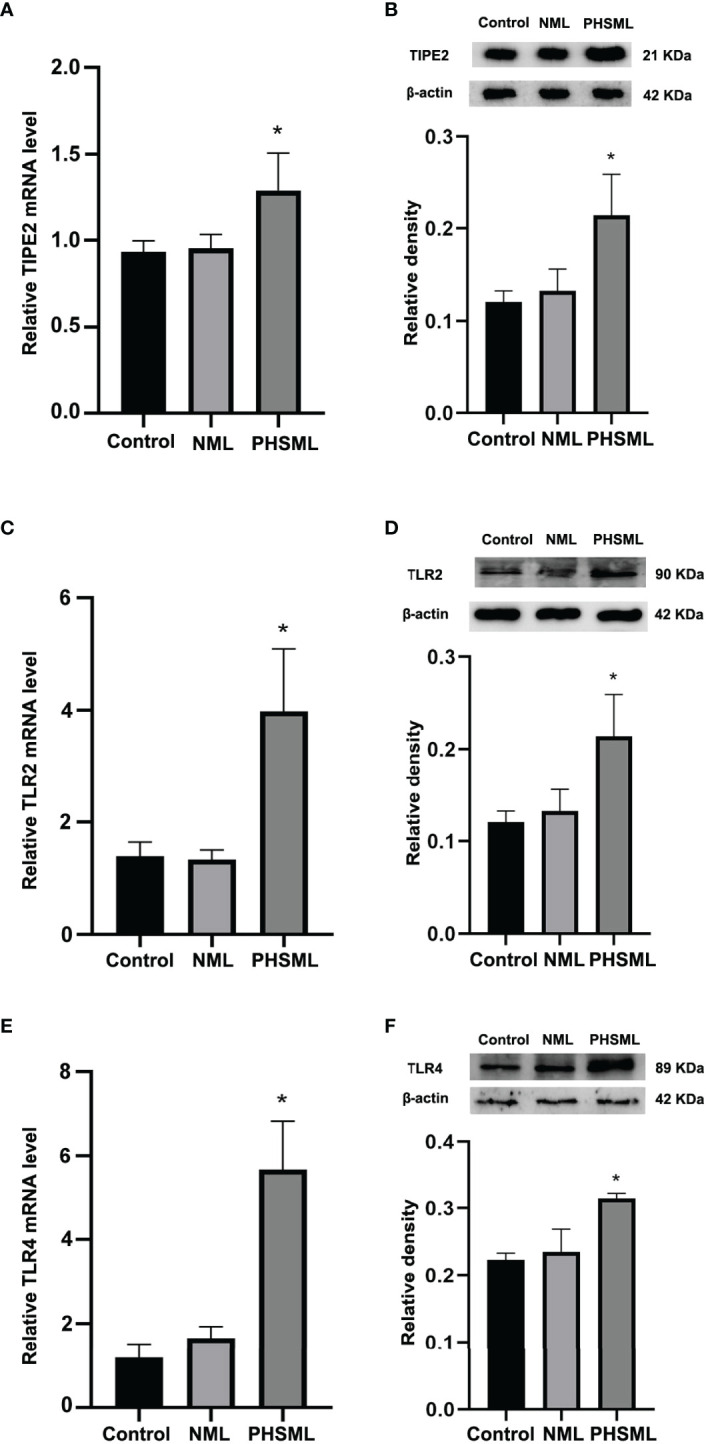
PHSML increased the expression level of TIPE2, TLR2 or TLR4 in CD4^+^T derived from WT mice. **(A)** The mRNA expressions of TIPE2 in CD4^+^T lymphocytes following stimulation with PHSML. **(B)** The protein expressions of TIPE2 in CD4^+^T lymphocytes following stimulation with PHSML. Compared to the Control group, PHSML increased the level of TIPE2. **(C)** The mRNA expressions of TLR2 in CD4^+^T lymphocytes following stimulating of PHSML. **(D)** The protein expressions of TLR2 in CD4^+^T lymphocytes following stimulating of PHSML. **(E)** The mRNA expressions of TLR4 in CD4^+^T lymphocytes following stimulating of PHSML. **(F)** The protein expressions of TLR4 in CD4^+^T lymphocytes following stimulating of PHSML. Compared to the NML group, PHSML increased the level of TLR2 and TLR4. Data are presented as mean ± SE (n = 3). *P < 0.05 vs control and NML groups.

To investigate the effect of PHSML on the expression of TIPE2 in WT CD4^+^T cells, qRT-PCR and Western blotting were used to detect the mRNA and protein expression levels of TIPE2 in WT CD4^+^T cells stimulated with PHSML or NML, respectively. Compared with the control group, there was no statistical significance in the expression levels of TIPE2 mRNA in NML group (P > 0.05), while TIPE2 mRNA expression levels of WT CD4^+^T were significantly elevated in PHSML-stimulated group (P < 0.05) (**Figure 1A**). Similar results were observed for TIPE2 protein expression (P < 0.05) (**Figure 1B**). Meanwhile, we investigated the effect of PHSML on the expression of TLR2 and TLR4 in WT CD4^+^ T cells. Quantitative RT-PCR and Western blot results showed that the expression of TLR2 and TLR4 were statistically significantly increased after PHSML stimulation (P < 0.05) (**Figures 1C–F**), consistent with the expression of TIPE2.

### Role of TLR2/TLR4 Mediated TIPE2 in PHSML-Induced Proliferation Deficiency of CD4^+^ T Cells

**Figure 2:**
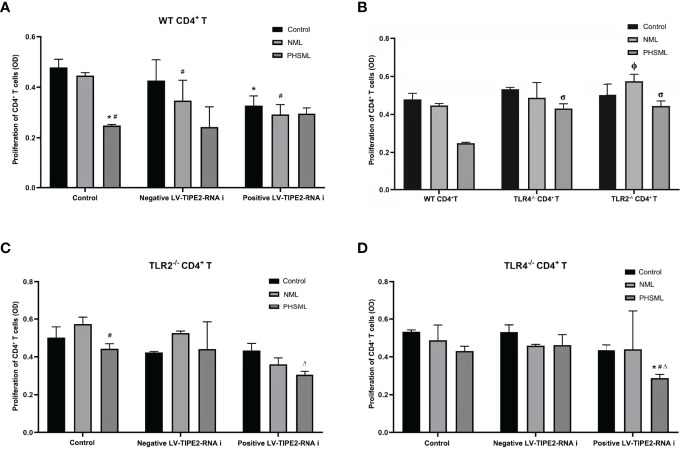
Role of TLR2/TLR4 Mediated TIPE2 in the proliferation of WT CD4^+^T lymphocytes reduced by PHSML. **(A)** The effect of TIPE2 expression on the proliferation of CD4^+^T cells induced by PHSML *in vitro*. **(B)** The effect of TLR2/TLR4 expression on the proliferation of CD4^+^T cells induced by PHSML *in vitro*. **(C)** The effect of TIPE2 and TLR2 expression on the proliferation of CD4+T cells induced by PHSML *in vitro*. **(D)** The effect of TIPE2 and TLR4 expression on the proliferation of CD4^+^T cells induced by PHSML *in vitro*. Data expressed as the mean ± SE (n = 6). *P < 0.05 vs control treated control group, #P < 0.05 vs NML treated control group, ΔP < 0.05 vs PHSML treated control group, φP < 0.05 vs NML treated WT CD4^+^T group, σP < 0.05 vs PHSML treated WT CD4^+^T group.

After transfection of lentivirus carrying interference of TIPE2 sequence (shRNA), the proliferation of WT CD4^+^T cells stimulated with PHSML or NML was measured by the CCK8 method. The results revealed that PHSML significantly reduced the proliferation capacity of WT CD4^+^ T cells, while silencing the expression of TIPE2 in WT CD4^+^ T cells attenuated the inhibitory effect of PHSML on CD4^+^ T cell proliferation (**Figure 2A**).

To investigate the role of TLR2 or TLR4 in PHSML-induced proliferation deficiency of CD4^+^ T cells, the proliferation of WT CD4^+^ T, TLR2^-/-^CD4^+^ T cells and TLR4^-/-^CD4^+^ T cells was examined with CCK8 assay. The results revealed that PHSML significantly decreased the proliferation of WT CD4^+^ T cells, while lack of TLR2 or lack of TLR4 attenuated the inhibitory effect of PHSML on the proliferation of CD4^+^ T cells (**Figure 2B**).

For the purpose of exploring the relationship between TIPE2 and TLR2/TLR4 in the ability of PHSML to reduce the proliferation CD4^+^ T cells, lentiviral carrying interference of TIPE2 sequence (shRNA) was used to transfect TLR2^-/-^CD4^+^ T cells or TLR4^-/-^CD4^+^ T to silence TIPE2 gene expression in the present study. Then PHSML or NML was given for stimulation, a blank control group was set up at the same time, and the proliferation capacity of CD4^+^ T lymphocytes in each group was detected by the CCK8 method. It was found that lack of TLR2 in CD4^+^ T cells ameliorated the inhibitory effect of PHSML on the proliferative function of CD4^+^ T cells, but silencing the expression of TIPE2 in TLR2^-/-^ CD4^+^ T cells, PHSML reduced the proliferative capacity of CD4^+^ T cells instead (**Figure 2C**), which indicating that either lack of TLR2 or low expression of TIPE2 relieved the reduced proliferation capacity of CD4^+^ T cells caused by PHSML. But the coexistence of lack of TLR2 and low expression of TIPE2 eliminated this beneficial effect. These results suggest that both TIPE2 and TLR2 were involved in the regulation of the proliferation capacity of CD4^+^ T cells by PHSML, and that TIPE2 may regulate proliferation dysfunction of CD4^+^ T cells caused by PHSML through TLR2.

It was found that lack of TLR4 in CD4^+^ T cells was beneficial to improve the inhibitory effect of PHSML on the proliferative function of CD4^+^ T cells. But by silencing expression of TIPE2 in TLR4^-/-^ CD4^+^ T cells, PHSML reduced the proliferative capacity of CD4^+^ T cells instead (**Figure 2D**), indicating that either lack of TLR4 or low TIPE2 expression alleviated the low proliferation capacity of CD4^+^ T cells caused by PHSML. However, the coexistence of lack of TLR4 and low expression of TIPE2 eliminated this beneficial effect, suggesting that both TIPE2 and TLR4 are involved in the process of regulating proliferative capacity of CD4^+^ T cells by PHSML, and that TIPE2 may play a regulatory role through TLR4.

### Role of TIPE2 in the Expression of CD25 on CD4^+^ T Cells Activated by PHSML

**Figure 3:**
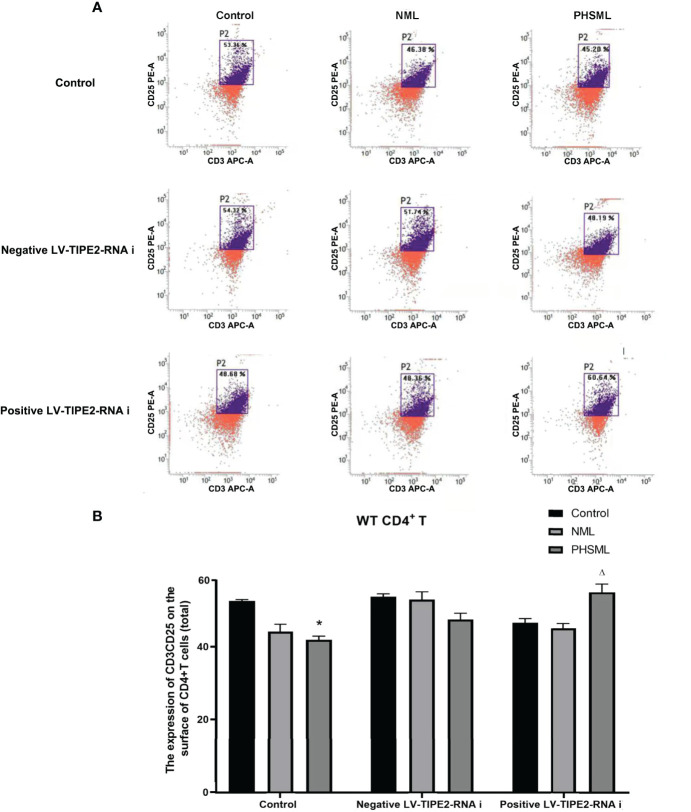
The expression of CD25 on CD4^+^T lymphocytes from WT mice infected with TIPE2 shRNA following by the stimulation with PHSML detected by flow cytometry. **(A)** Representative images. **(B)** The percentage of CD4^+^T cells expressing CD25 is expressed as the mean ± SE and is shown in a bar graph. Data are derived from three independent experiments. *P < 0.05 vs control treated control group, ΔP < 0.05 vs PHSML treated control group.

WT CD4^+^ T cells were transfected with lentivirus carrying a TIPE2-interfering fragment (shRNA) to silence gene expression of TIPE2. Then WT CD4^+^T cells stimulated with different types of mesenteric lymph (NML, PHSML). And flow cytometry was used to detect the expression of CD25 (IL-2R) in CD4^+^ T cells in each group (**Figure 3**). PHSML significantly decreased the expression of CD25 in CD4^+^ T cells, and after silencing the expression of TIPE2 in CD4^+^ T cells, the expression of CD25 in CD4^+^ T cells was upregulated, indicating that PHSML suppressed the activation of CD4^+^ T cells. Moreover, low expression of TIPE2 was beneficial to improve the suppressed state of CD4^+^ T cells activation caused by PHSML, which suggesting that TIPE2 is an important molecule that regulates the dysfunction of CD4^+^ T cells activation caused by PHSML.

### Role of TLR2/TLR4 in the Expression of CD25 on CD4^+^ T Cells Activated by PHSML

**Figure 4:**
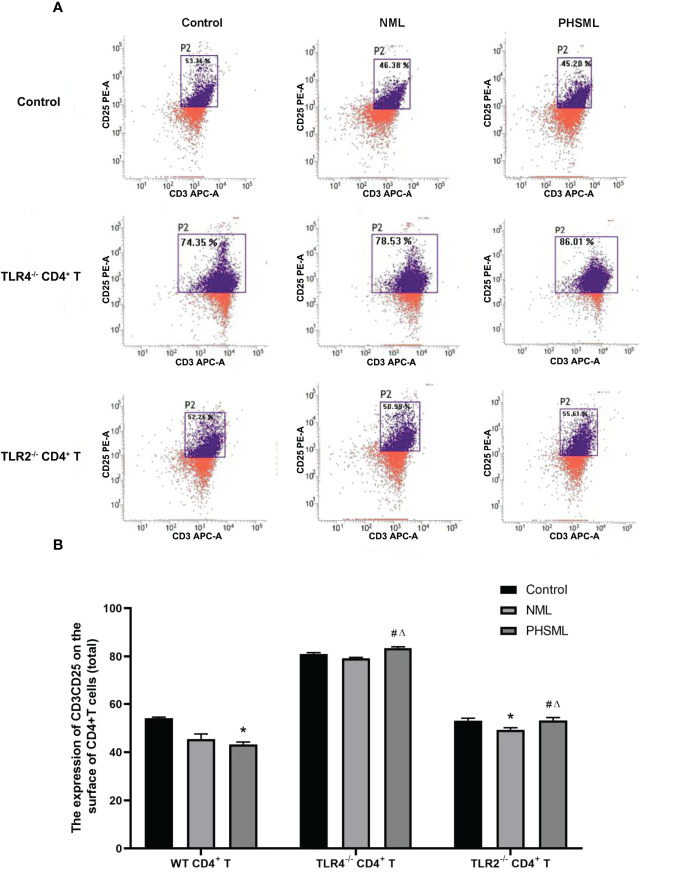
The expression of CD25 on CD4^+^T lymphocytes from WT, TLR2^-/-^ or TLR4^-/-^ mice stimulated with PHSML detected by flow cytometry. **(A)** Representative images. **(B)** The percentage of CD4^+^T cells expressing CD25 is expressed as the mean ± SE and is shown in a bar graph. Data are derived from three independent experiments. *P < 0.05 vs control treated WT CD4^+^T group, #P < 0.05 vs NML treated WT CD4^+^T group, ΔP < 0.05 vs PHSML treated WT CD4^+^T group.

In order to explore the role of TLR2 or TLR4 in the activation of CD4^+^ T cells by PHSML and to further elucidate the importance and significance of TLR2 or TLR4 in immune dysfunction due to hemorrhagic shock, immunomagnetic bead sorting methods were used to isolate and culture CD4^+^ T cells of different (WT, TLR2^-/-^, TLR4^-/-^) mice which stimulated with PHSML or NML, and expression of CD25 (IL-2R) upon activated CD4^+^ T cells was examined with flow cytometry (**Figure 4**). PHSML significantly decreased the expression of CD25 in WT CD4^+^ T cells. Deletion of TLR4 significantly enhanced the expression of CD25 in CD4^+^ T cells in each stimulation group. Compared with WT CD4^+^ T cells, lack of TLR2 significantly increased expression of CD25 in CD4^+^ T cells with stimulation of PHSML. And either TLR4 or TLR2 was involved in the PHSML-regulated activation process of CD4^+^ T cells. However, the involvement of TLR4 in the process of the activation of CD4^+^ T cells caused by PHSML was seemingly more predominantly important in the development and progression of hemorrhagic shock.

### Role of TLR2-Mediated TIPE2 in the Expression of CD25 on CD4^+^ T Cells Activated by PHSML

**Figure 5:**
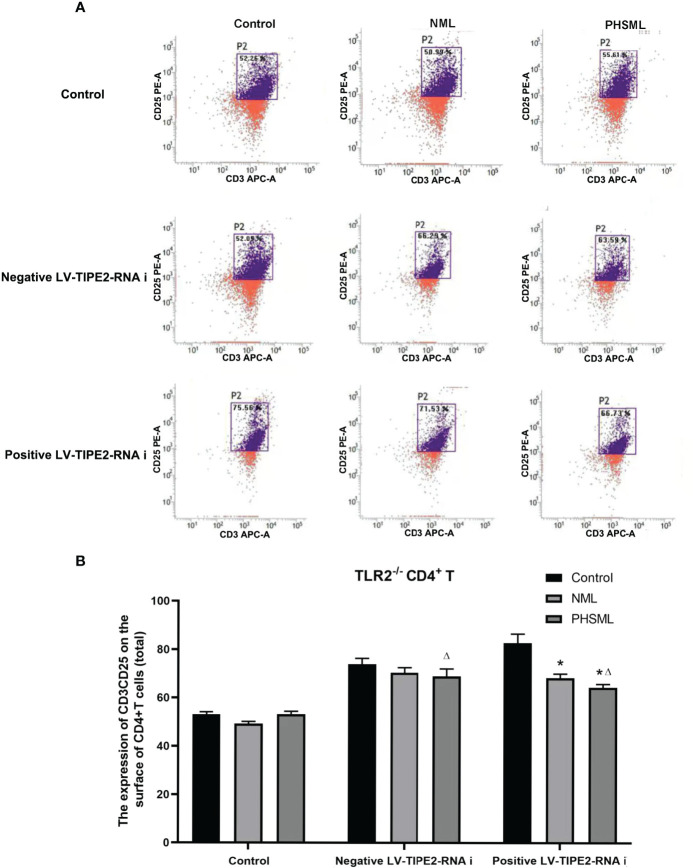
The expression of CD25 on TLR2^-/-^ CD4^+^T lymphocytes infected with TIPE2 shRNA following by the stimulation with PHSML detected by flow cytometry. **(A)** Representative images. **(B)** The percentage of CD4^+^T cells expressing CD25 is expressed as the mean ± SE and is shown in a bar graph. Data are derived from three independent experiments. *P < 0.05 vs control treated control group, ΔP < 0.05 vs PHSML treated control group.

In order to explore the interaction between TIPE2 and TLR2 in the activation CD4^+^ T cells caused by PHSML, lentiviral carrying interference of TIPE2 sequence (shRNA) was used to transfect TLR2^-/-^CD4^+^ T cells to silence gene expression of TIPE2 in this experiment. Then PHSML or NML was given for stimulation, a blank control group was set up at the same time. Flow cytometry was used to detect expression of CD25 in CD4^+^ T lymphocytes in each group (**Figure 5**). The above results showed that lack of TLR2 or low expression of TIPE2 in WT CD4^+^ T cells improved the inhibitory state of activated CD4^+^ T cells caused by PHSML. However, the simultaneous presence of lack of TLR2 and low expression of TIPE2 still upregulated expression of CD25. This suggests that both TIPE2 and TLR2 are involved in the regulation of the activation of CD4^+^ T cells caused by PHSML, while the interaction between TIPE2 and TLR2 needs to be further investigated.

### Role of TLR4-Mediated TIPE2 in the Expression of CD25 on CD4^+^ T Cells Activated by PHSML

**Figure 6:**
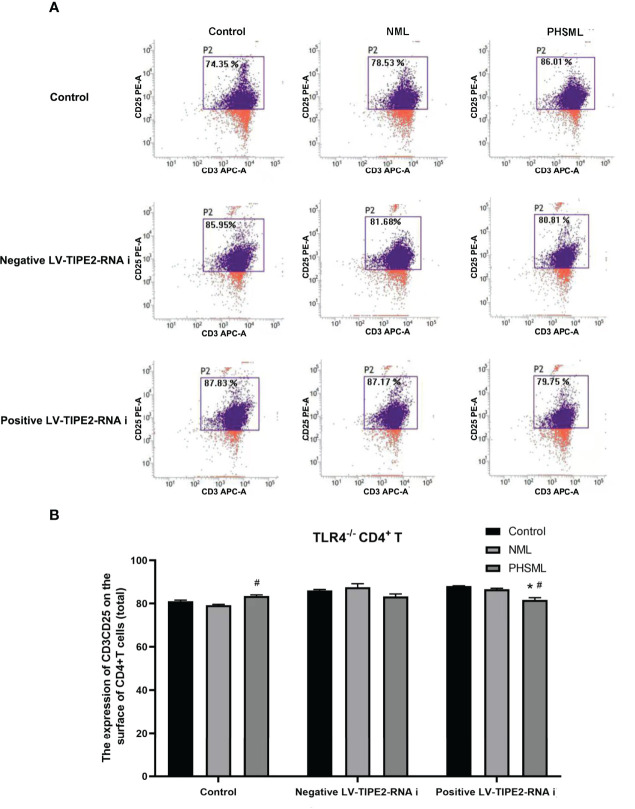
The expression of CD25 on TLR4^-/-^ CD4^+^T lymphocytes infected with TIPE2 shRNA following by the stimulation with PHSML detected by flow cytometry. **(A)** Representative images. **(B)** The percentage of CD4^+^T cells expressing CD25 is expressed as the mean ± SE and is shown in a bar graph. Data are derived from three independent experiments. *P < 0.05 vs control treated control group, #P < 0.05 vs PHSML treated control group.

In order to investigate the interaction between TIPE2 and TLR4 in the activation of CD4^+^ T cells by PHSML, lentiviral carrying interference of TIPE2 sequence (shRNA) was used to transfect TLR4^-/-^ CD4^+^ T cells to silence gene expression of TIPE2 in this experiment. Then PHSML or NML was given for stimulation, a blank control group was set up at the same time. Flow cytometry was applied to detect expression of CD25 in CD4^+^ T lymphocytes in each group (**Figure 6**). It was found that deletion of TLR4 in CD4^+^ T cells was beneficial in ameliorating the suppressed activation function of CD4^+^ T cells caused by PHSML. But by silencing the expression of TIPE2 in TLR4^-/-^ CD4^+^ T cells, PHSML decreased the expression of CD25 in CD4^+^ T cells, indicating that either deletion of TLR4 or low expression of TIPE2 ameliorated the PHSML-induced low expression status of CD25 in CD4^+^ T cells. However, the simultaneous presence of deletion of TLR4 and low expression of TIPE2 eliminated this beneficial effect, suggesting that both TIPE2 and TLR4 are involved in the regulation of the activation of CD4^+^ T cells by PHSML, suggesting that TIPE2 may be involved in the regulation of the CD4^+^ T cells activation by PHSML through TLR4.

### Effect of TLR2/TLR4-Mediated TIPE2 on the Levels of Cytokines Secreted by CD4^+^ T Cells Stimulated With PHSML

Different cytokines represent the direction of differentiation of CD4^+^ T cells into effector cells with different immune functions. IFN-ɣ is mainly secreted by Th1 cells and mediates cellular immunity. IL-4 is mainly secreted by Th2 cells and mediates humoral immunity. IL-10 is mainly secreted by T cells, which is regarded as an anti-inflammatory cytokine. We further investigated the role of TLR2/TLR4-mediated TIPE2 in the secretion levels of cytokines in WT CD4^+^ T cells stimulated with PHSML. The secretion levels of cytokines in WT CD4^+^ T, TLR2^-/-^CD4^+^ T, or TLR4^-/-^CD4^+^ T cells stimulated with PHSML were measured by ELISA, respectively. Lentiviral carrying Interference of TIPE2 sequence (shRNA) was used to transfect WT CD4^+^ T, TLR2^-/-^CD4^+^ T, or TLR4^-/-^CD4^+^ T cells to silence gene expression of TIPE2. Then PHSML or NML was given for stimulation, a blank control group was set up at the same time. And the secretion level of different cytokines in the culture supernatant of each experimental group was measured by ELISA.

**Figure 7:**
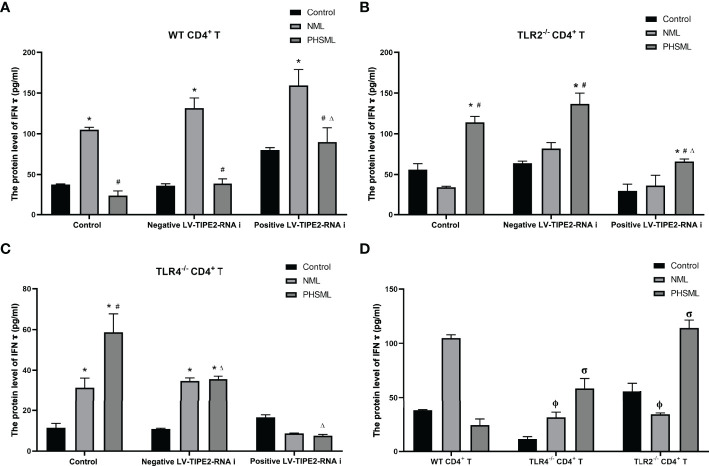
The changes of IFN-γ production in CD4^+^T cells isolated from various mice. **(A)** The production of IFN-γ in cultural supernatant of CD4^+^T cells from WT mice infected with TIPE2 shRNA following by the stimulation of PHSML detected by ELISA; **(B)** The production of IFN-γ in cultural supernatant of CD4^+^T cells from TLR2^-/-^ mice infected withTIPE2 shRNA following by the stimulation with PHSML detected by ELISA; **(C)** The production of IFN-γ in cultural supernatant of CD4^+^T cells from TLR4^-/-^ mice infected with TIPE2 shRNA following by the stimulation with PHSML detected by ELISA; **(D)** The production of IFN-γ in cultural supernatant of CD4^+^T cells from WT, TLR2^-/-^ or TLR4^-/-^ mice stimulated with PHSML detected by ELISA, respectively. Data are expressed as the mean ± SE (n = 6). *P < 0.05 vs control treated control group, #P < 0.05 vs NML treated control group, ΔP < 0.05 vs PHSML treated control group, φP < 0.05 vs NML treated WT CD4^+^T group, σP < 0.05 vs PHSML treated WT CD4^+^T group.

The results showed that PHSML significantly decreased the secretion level of IFN-γ, deletion of TLR2 or deletion of TLR4 significantly increased the secretion level of IFN-γ in CD4^+^ T cells stimulated with PHSML. Low expression of TIPE2 also significantly increased the secretion level of IFN-γ in WT CD4^+^ T cells stimulated with PHSML, while coexisting of deletion of TLR2 or deletion of TLR4 with low expression of TIPE2 eliminated this beneficial effect (**Figure 7**).

**Figure 8:**
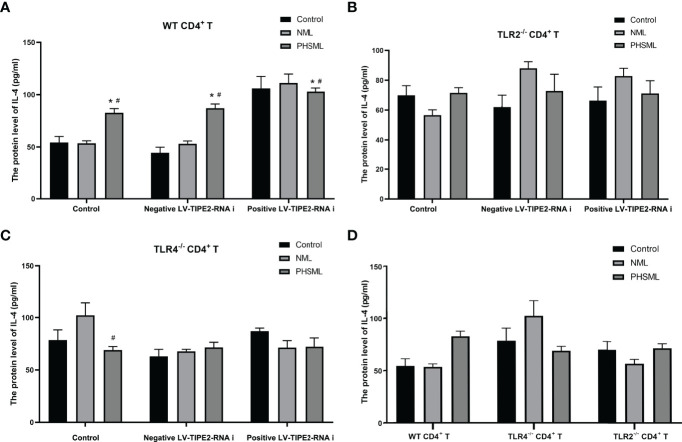
The changes of IL-4 production in CD4^+^T cells isolated from various mice. **(A)** The production of IL-4 in cultural supernatant of CD4^+^T cells from WT mice infected with TIPE2 shRNA following by the stimulation with PHSML detected by ELISA; **(B)** The production of IL-4 in cultural supernatant of CD4^+^T cells from TLR2^-/-^ mice infected with TIPE2 shRNA following by the stimulation with PHSML detected by ELISA; **(C)** The production of IL-4 in cultural supernatant of CD4^+^T cells from TLR4^-/-^ mice infected with TIPE2 shRNA following by the stimulation with PHSML detected by ELISA; **(D)** The production of IL-4 in cultural supernatant of CD4^+^T cells from WT, TLR2^-/-^ or TLR4^-/-^ mice stimulated with PHSML detected by ELISA, respectively. Data are expressed as the mean ± SE (n = 6). *P < 0.05 vs control treated control group, #P < 0.05 vs NML treated control group.

However, the results showed that PHSML significantly increased the secretion level of IL-4 from WT CD4^+^ T cells, deletion of TLR4 significantly decreased the secretion level of IL-4 in CD4^+^ T cells after stimulated with PHSML, and deletion of TLR2 had no significant effect on the secretion level of IL-4 in CD4^+^ T cells after stimulation of PHSML. Low expression of TIPE2 had no significant effect on the secretion level of IL-4 in WT CD4^+^ T cells after stimulated with PHSML. And coexisting of deletion of TLR2 or deletion of TLR4 with low expression of TIPE2 had no significant effect on the level of IL-4 in PHSML-activated CD4^+^ T cells (**Figure 8**).

**Figure 9:**
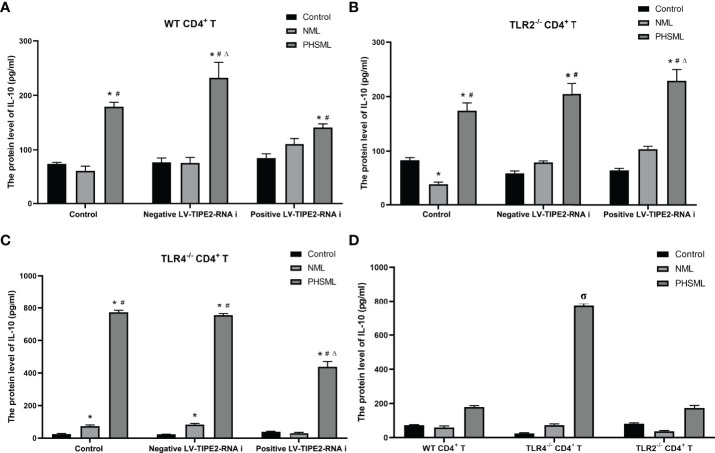
The changes of IL-10 production in CD4^+^T cells isolated from various mice. **(A)** The production of IL-10 in cultural supernatant of CD4^+^T cells from WT mice infected with TIPE2 shRNA following by the stimulation with PHSML; **(B)** The production of IL-10 in cultural supernatant of CD4^+^T cells from TLR2^-/-^ mice infected with TIPE2 shRNA following by the stimulation with PHSML detected by ELISA; **(C)** The production of IL-10 in cultural supernatant of CD4^+^T cells from TLR4^-/-^ mice infected with TIPE2 shRNA following by the stimulation with PHSML detected by ELISA; **(D)** The production of IL-10 in cultural supernatant of CD4^+^T cells from WT, TLR2^-/-^ or TLR4^-/-^ mice stimulated with PHSML detected by ELISA, respectively. Data are expressed as the mean ± SE (n = 6). *P < 0.05 vs control treated control group, #P < 0.05 vs NML treated control group, ΔP < 0.05 vs PHSML treated control group, σP < 0.05 vs PHSML treated WT CD4^+^T group.

The results showed that the secretion level of IL-10 from WT CD4^+^ T cells was significantly increased by PHSML, deletion of TLR4 significantly increased the secretion level of IL-10 in CD4^+^ T cells after stimulation of PHSML, and deletion of TLR2 had no significant effect on the secretion level of IL-10 in CD4^+^ T cells after stimulation of PHSML. Low expression of TIPE2 decreased the secretion level of IL-10 in CD4^+^ T cells after stimulation of PHSML, deletion of TLR2 along with low expression of TIPE2 increased the secretion level of IL-10 in CD4^+^ T cells stimulated with PHSML, and deletion of TLR4 along with low expression of TIPE2 decreased the secretion level of IL-10 in CD4^+^ T cells after stimulation of PHSML (**Figure 9**). Data of cytokines are listed in **Supplementary Table 1**.

## Discussion

Immune dysfunction due to hemorrhagic shock has been widely investigated as a research hotspot because it can cause systemic inflammation and multi-organ failure (28–31). However, the mechanisms of disorder of cellular immune function induced by hemorrhagic shock are not fully understood. It is widely believed that PHSML reflux due to impaired mesenteric barrier and dysfunctional mesenteric lymph node in the body after hemorrhagic shock is strongly associated with distal tissue damage and decrease host resistance to microbial infection (11, 32–34). It has been reported that after mesenteric lymph node structure and function are damaged, the Th1/Th2 subsets of CD4^+^ T cells are dysregulated, and the proliferation and differentiation of CD4^+^ T cells are interrupted, thus leading to immune imbalance in the body (35–37). New studies have found that lymphoid tissue inducer (LTi) cells in the mesenteric lymph nodes interact with CD4^+^ T cells to maintain the normal immune effects of CD4^+^ T cells in the intestinal microenvironment, while the release of LTi cells inhibits CD4^+^ T cells function (38). However, the role of PHSML in CD4^+^ T cells activation remains to be further studied.

In this study, we found that PHSML triggers the suppression of immune function by upregulating TIPE2 associated with TLR2/TLR4 in CD4^+^ T cells. Our study provides a new perspective on the potential mechanism of PHSML triggering cellular immune dysfunction and provides a possible intervention target for maintaining immune homeostasis.

TIPE2 is a negative immunoregulatory molecule first identified in autoimmune encephalomyelitis (17), which plays an immunomodulatory role by delivering different signals to pattern recognition receptors on the cell surface, including TLRs (17, 39). TIPE2 is mainly involved in the generation and regulation of innate immunity and the subsequent initiation of adaptive immune responses (40). TIPE2 receives signals from exogenous pathogen-associated molecular pattern (PAMP) and damage-associated molecular pattern (DAMP) to distinguish between “non-self” and “self” components to identify different microorganisms and endogenous ligands (41). An increasing number of studies have shown that abnormal expression of TIPE2 and TLR is associated with the pathogenesis of inflammatory diseases and injuries (42–44). For example, the expression of TIPE2 was significantly down-regulated in lung tissues of mice with acute lung injury (43), accompanied by upregulation of TLR and secretion of a large number of cytokines (45). In hepatitis mice, TIPE2 overexpression and TIPE2 knockdown alleviated and exacerbated steatosis and inflammation, respectively, and the expression level of TIPE2 was negatively correlated with TAK1 which is a downstream molecule of TLR (46). A similar phenomenon was observed in Alzheimer’s mice (47), which may be related to the cellular immunosuppression induced by elevated TIPE2 to reduce the inflammatory response. At the same time, we observed that TIPE2 is often closely associated with expression of TLR2/TLR4 in TLR in inflammatory diseases (23–25). Thus, we measured TLR2/TLR4 related indicators in CD4^+^ T cells and observed the effect of the deletion of TLR2/TLR4 on activation of CD4^+^ T cells. In the present study, the results showed that PHSML increased the mRNA and protein expression levels of TIPE2, TLR2 and TLR4 in WT CD4^+^ T cells. Notably, in general inflammatory mouse models, TIPE2 accomplishes the corresponding immunological effects by negatively regulating TLR, whereas the expression of TIPE2 and TLR2/TLR4 in this study seems to be positively correlated, and our previous study showed that TIPE2 was also proportional to the expression levels of downstream molecules of TLR (32). This may be related to PHSML-induced CD4^+^ T cells immune dysfunction, resulting in TIPE2 losing normal immune regulatory function, and the related mechanism needs to be further studied.

In view of the important role of TIPE2 in maintaining immune homeostasis, an increasing number of studies have used TIPE2 as a therapeutic target for inflammatory diseases by silencing TIPE2 (48) or constructing overexpression vectors (43) to upregulate or inhibit the intensity of the immune response. To combat septic shock, it has been reported that the expression of TIPE2 is upregulated to reduce excessive inflammatory responses of macrophages, neutrophils, dendritic cells, T cells and B cells and to maintain hemostasis (17, 49). However, the role of TIPE2 in the shock treatment, especially immunosuppression after hemorrhagic shock, has not been reported. In our experiments, TIPE2 was used as an intervention target to modulate PHSML-induced cellular immunosuppression, providing new insights into the regulation of immune disorders after hemorrhagic shock.

Many previous studies have shown that a reduction in the number of CD4^+^ T cells in shock patients induces severe immunosuppression and is associated with high mortality and secondary infection rates (50). The results of this study show that PHSML inhibits the proliferation of CD4^+^ T cells, which is consistent with our previous studies (15). Low expression of TIPE2 and deletion of TLR2 or TLR4 ameliorated the suppressive effect of PHSML, which was lost when deletion of TLR2 or TLR4 coexisted with low expression of TIPE2. These results suggest that TIPE2 may regulate the proliferation of CD4^+^ T cells through TLR2/TLR4, and that using TLR2, TLR4, or TIPE2 as therapeutic targets can improve the inhibition of PHSML on the proliferation of CD4^+^ T cells.

CD25 is an important activation marker on the surface of CD4^+^ T cells (51), and the upregulation of CD25 is associated with the activation process of CD4^+^ T cells. Our study showed that PHSML significantly reduced the expression of CD25 in CD4^+^ T cells compared with NML group. The expression of CD25 in the positive LV-TIPE2-RNAi group of WT mice and the PHSML group of TLR2^-/-^ and TLR4^-/-^ mice was increased compared with the corresponding control group, and the deletion of TLR4 played a more significant role in the activation of CD4^+^ T cells than the deletion of TLR2. However, the activation of CD4^+^ T cells with both TLR4 deletion and low expression of TIPE2 was altered compared with normal expression of TIPE2, while there was no such change in both the deletion of TLR2 and low expression of TIPE2. This suggests that TIPE2 may be involved in the regulation of CD4^+^ T cells activation by PHSML through TLR4 rather than TLR2.

It has been shown that the Th1/Th2 ratio in the Th subpopulation is imbalanced and preferentially develops toward the Th2 subtype after cellular immune disorders in patients with hemorrhagic shock (52). It is well known that IFN-γ is mainly secreted by Th1 cells and mediates cellular immunity, whereas IL-4 and IL-10 are mainly secreted by Th2 cells, mediating humoral immunity and inhibiting Th1 cell proliferation. Our results showed that PHSML decreased the secretion of IFN-γ and increased the level of IL-4 secretion in CD4^+^ T cells, which is consistent with our previous studies (15). In addition, PHSML significantly increased the secretion level of IL-10, which was associated with immunosuppression due to hemorrhagic shock (53). After silencing TIPE2, we observed increased levels of IFN-γ secretion and decreased levels of IL-10 secretion, suggesting that TIPE2 is associated with the switching of Th cell subtype due to PHSML. Further studies revealed that TLR4 rather than TLR2 is involved in TIPE2-mediated Th1/Th2 imbalance induced by PHSML. Notably, TLR4 deletion did not downregulate IL-10 secretion levels, but did the opposite. This may be related to the significant upregulation of CD25 caused by TLR4 deletion in this study, since there is substantial evidence that the elevation of IL-10 and CD25 in CD4^+^ T cells tends to coexist and be positively correlated (54, 55).

Like other immunomodulatory molecules, TIPE2 relies on a complex network of signaling pathways to perform immune function. The interaction between TIPE2 and TLR has been proved to be closely linked to the maintenance of immune homeostasis (18, 19). Whether silencing of TIPE2 affects TLR2/TLR4 and its downstream important signaling molecules such as TRIF-TRAF3 axis, MyD88-TRAF6 axis and whether it is related to cross-talk between TLR2 and TLR4 pathways is still unclear, which will be the focus of our future studies.

In conclusion, our study shows that PHSML inhibits CD4^+^ T cell proliferation, surface molecule expression and cytokine secretion *via* the TLR2/TLR4-mediated TIPE2 pathway. The new mechanism of TIPE2 inducing cellular immune disorders provides new strategy for elucidating the relevant molecular signaling mechanism, the treatment and prognosis of hemorrhagic shock.

## Data Availability Statement

The original contributions presented in the study are included in the article/**Supplementary Material**. Further inquiries can be directed to the corresponding authors.

## Ethics Statement

The animal study was reviewed and approved by Animal Care Committee of Hebei North University.

## Author Contributions

H-BD, S-BJ, HZ, ZW, and Y-XG recorded and analyzed the general data. S-BJ wrote the original manuscript draft. H-BD and L-MZ were responsible for hemorrhagic shock model. Z-AZ, J-YZ, and PW contributed to editing and revisions. H-BD and S-BJ contributed equally to this work. Z-GZ, L-NJ, and C-YN were responsible for the general conception and design of the study. All authors contributed to the article and approved the submitted version.

## Funding

This work was supported by grants from the National Natural Science Foundation of China (No. 81701963), the Natural Science Foundation of Hebei Province (H2020405023).

## Conflict of Interest

The authors declare that the research was conducted in the absence of any commercial or financial relationships that could be construed as a potential conflict of interest.

## Publisher’s Note

All claims expressed in this article are solely those of the authors and do not necessarily represent those of their affiliated organizations, or those of the publisher, the editors and the reviewers. Any product that may be evaluated in this article, or claim that may be made by its manufacturer, is not guaranteed or endorsed by the publisher.

## Abbreviations

PHSML, post-hemorrhagic shock mesenteric lymph; NML, normal mesenteric lymph; TIPE2, Tumor necrosis factor α induced protein 8 like-2; TLR2, Toll-like receptors 2; TLR4, Toll-like receptors 4.
