# Supplementary material for: TLR2/TLR4-Enhanced TIPE2 Expression Is Involved in Post-Hemorrhagic Shock Mesenteric Lymph-Induced Activation of CD4+T Cells
Source: Front Immunol. 2022 Apr 29;13:838618. doi: 10.3389/fimmu.2022.838618 (PMC9101470; doi:10.3389/fimmu.2022.838618)
Supplement: Supplementary file 1 [file DataSheet_1.doc]

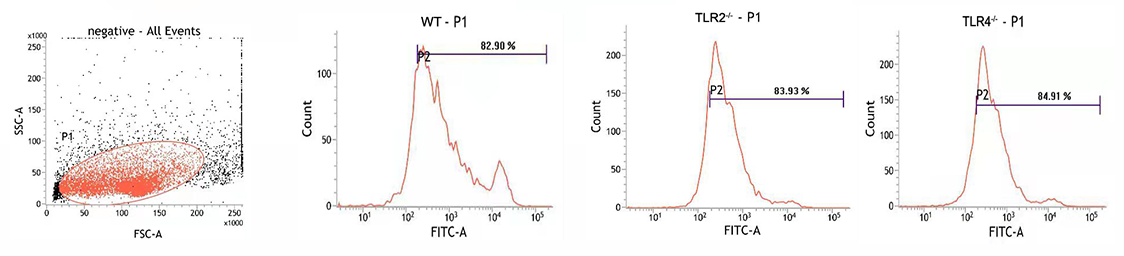


**Supplemental Fig 1**. LV-TIPE2-RNAi transfection efficiency. Flow cytometry analysis demonstrated that transfection rate of LV-TIPE2-RNAi in CD4+ T lymphocytes was 82.9%.


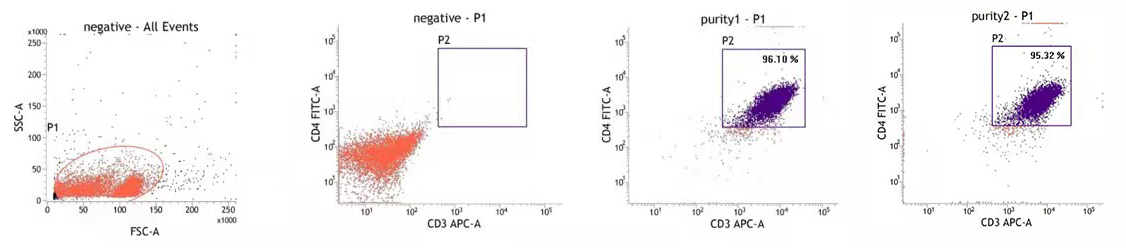


**Supplemental Fig 2**. The purity of CD4^+^T cells. Flow cytometry analysis demonstrated that cells contained > 90% CD4^+^T lymphocytes.

**Supplemental Table 1**. The data of cytokines in CD4+T cell (mean±SE, n=6)
